# The Management of Patients with Cataracts and Medically Uncontrolled Glaucoma

**Published:** 2014

**Authors:** Rahat Husain

**Affiliations:** Singapore Eye Research Institute and Singapore National Eye Centre, Singapore

**Keywords:** Cataract, Uncontrolled Glaucoma, Trabeculectomy, Cyclophotocoagulation, IOP, Bleb, Complication

## Abstract

Trabeculectomy surgery has been shown to lower intraocular pressure and is the most commonly performed glaucoma procedure worldwide. However, giving a patient a ‘bleb for life’ is not without consequences and the failure of trabeculectomy to control IOP in the long term is well documented. In some instances, such as in patients with exfoliative glaucoma or primary angle closure glaucoma, cataract surgery alone can often lower IOP to acceptable levels. Cataract surgery in these instances can sometimes be combined with procedures such as goniosynechialysis or endoscopic cyclophotocoagulation which may provide additional IOP lowering. Such surgery has the distinct advantage of avoiding conjunctival incisions, so that subsequent trabeculectomy, if required, is more likely to be successful. In any case, it is preferable to perform trabeculectomy in a pseudophakic eye for several reasons. If trabeculectomy is performed in a phakic eye, patients should be warned that subsequent cataract is likely and if cataract surgery is performed it is preferable to wait at least a year or more after the trabeculectomy to reduce the risk of bleb failure. Combined phacotrabeculectomy should be reserved for end-stage glaucoma in most cases, in order to reduce the risk of ‘wipe-out’.

## INTRODUCTION

Patients with cataracts and glaucoma are common in hospital clinics. There has been much published in recent years on how best to manage these subjects (1-4). This review will attempt to summarise the evidence and help the general ophthalmologist make informed decisions on management based on the available data. 

 As this review is intended for the general ophthalmologist, discussion will exclude aqueous drainage device implantation, non-penetrating glaucoma surgery and devices and procedures such as the Ex-Press shunt, the istent, canaloplasty etc. That is not because these procedures are without merit, but most general ophthalmologists, and indeed, many glaucoma specialists, will be less familiar with them. In addition, particularly for the latter procedures, long term data on efficacy and safety is pending. Essentially, in this context, glaucoma surgery refers to trabeculectomy.

 As with all fields in medicine, management will depend on several factors and will have to be tailored to the individual patient. Some factors that need to be assessed for all patients would be age, severity of visual field loss, intraocular pressure, rate of glaucomatous progression, type of glaucoma and degree of cataract growth. The aim of treatment is of course to improve quality of life which essentially means improving vision (cataract surgery) and slowing glaucomatous progression; these treatments must occur while causing the least discomfort and inconvenience to patients and by keeping complications and side effects to a minimum.

 There are 3 principles that are useful in guiding clinicians as to the best management for these patients. These are:

**1. Giving a patient a trabeculectomy bleb for life can be problematic and should be avoided if other options are available.**

**2. Trabeculectomy surgery is not always necessary in order to lower IOP.**

**3. If trabeculectomy is necessary, it is important to prime the eye to enable the best chance of success.**

 These 3 principles will be discussed further to provide evidence of their viability as treatment options and explore how they can help in making management decisions.

**Trabeculectomy surgery is associated with long-term problems and therefore should be avoided if possible.**

Modern cataract surgery is a relatively quick and safe procedure and therefore one can assume a good outcome in most cases. It is a one-off event, and if it is performed without complication, it is unlikely to result in further difficulties or morbidity (1-10).

 Glaucoma surgery, specifically trabeculectomy, is different. Even if the procedure is performed without complication, future morbidity often does occur. The most obvious of these is loss of IOP control, or trabeculectomy failure. Rates for this vary according to the population studied (5) and the definition of failure, but can be as high as 84% at 3 years (6). It could be argued that if the trabeculectomy fails, the patient goes back to ‘square one’ and, other than the inconvenience caused, no harm is done. However, it is the experience of many surgeons that the IOP after a failed trabeculectomy is higher than the IOP before trabeculectomy, even if drops are re-started. There is some experimental data supporting this observation (7,8) and it is postulated that diversion of aqueous via the sclerostomy to the sub-tenons space may compromise trabeculectomy meshwork functionality. There is good evidence that anterior chamber shallowing occurs after trabeculectomy and this lasts at least 5 years (9). This may result in mechanical closure of the angle (peripheral anterior synechiae or PAS), further compromising outflow via the conventional route. Therefore, to aver that a failed trabeculectomy constitutes ‘no harm done’ is likely to be incorrect

 Bleb-related infections can occur years after trabeculectomy. The Collaborative Initial Glaucoma Treatment Study (CIGTS) found rates of blebitis of 1.5% and bleb-related endophthalmitis of 1.1% at 5 years (10). Other studies have found higher rates (11,12). In the contemporary setting, use of anti-metabolites is ubiquitous, such that the NICE guidelines from UK recommend anti-metabolite use for all subjects undergoing trabeculectomy. To achieve lower IOPs, longer duration of antimetabolite use intraoperatively is often required, leading to thinner blebs and higher risk if infection (13). The pertinent fact is that risk of blebitis never goes away and patients with thin walled blebs will need to be aware of the risk of infection for the rest of their lives, something that cataract surgery patients, as an example, need not worry about. Many surgeons will advise patients that have had trabeculectomy to avoid swimming and yoga for example. The assumption that for most of patients attending glaucoma clinics these pastimes are not relevant (because of age) is condescending and probably untrue. Restricting these activities can have a significant effect on quality of life, no matter how well intentioned the advice.

 Dysesthesia is a common symptom reported following trabeculectomy and may indeed be under-reported as clinicians (and sometimes patients) tend to concentrate on IOP level – if IOP is low, the surgery is considered a success. In a prospective study of 97 subjects who had had trabeculectomy in one year, symptoms such as pain, discomfort, burning were significantly more common in the surgery eye compared to the fellow eye (dysesthesia score of 11.1 versus 3.4, p=0.005) (14). Similar findings were reported in the CIGTS where patients reported significantly more local eye symptoms in the eye which had had trabeculectomy compared to the eye randomized to medications (15).

 Ptosis, again from the CITGS, was found to occur in 12% of subjects in the trabeculectomy arm (16). A more recent study has found lid retraction to be a significant problem in some eyes that have had trabeculectomy (17). Both conditions can result in poor cosmesis and further surgery is sometimes required.

 In summary, although trabeculectomy is performed to lower IOP, and frequently does do so in the short to medium term, longer term results are less satisfactory. The surgery is associated with significant and relatively common complications.Following the dictum of ‘primum non nocere’ i.e. first do no harm, surgeons need to consider very carefully before performing trabeculectomy. The conclusion is that other procedures should be considered, if appropriate. This leads on to the second principle.

## Trabeculectomy surgery is often not necessary in order to lower IOP

Assuming that medical treatment is at a maximum tolerated level, there’s no doubt that trabeculectomy results in a lowering of the IOP, albeit not necessarily in the long term. Following the premise discussed above, are there any other ways to lower IOP without producing a trabeculectomy bleb? 

 In two specific instances, there is evidence that cataract surgery alone can lower IOP to satisfactory levels in a large proportion of cases. These are in patients with exfoliative glaucoma (XFG) and in patients with primary angle closure glaucoma (PACG). 

 Prevalence of XFG varies from region to region, with some evidence to suggest that it is more common in northern latitudes (18). In some countries, prevalence of XFG can account for more than half of cases of open angle glaucoma, and worldwide XFG is thought to account for 20-25% of open angle glaucoma (19). There is some evidence that performing cataract surgery in eyes with XFG results in reduced IOP, either alone (20) or if combined with trabecular aspiration (21,22). A large single surgeon series of phacoemulsification cataract surgery alone in eyes with exfoliation syndrome and XFG found a significant reduction in IOP at all-time points up to 7 years post-surgery (23). A later study published by the same group compared these findings with similar subjects that had undergone combined phacotrabeculectomy. They found the combined surgery group had lower IOP at 7 years (24). This suggests that in XFG patients, if low IOP is required, combined surgery may be preferable, but if a moderate reduction of IOP is all that is needed, cataract surgery alone may be sufficient.

 For patients with PACG, the evidence that cataract surgery reduces IOP is more extensive. Tham *et al*. have published a series of high quality prospective studies examining this topic. Comparing phacoemulsification to trabeculectomy in eyes with medically uncontrolled PACG, they found no significant difference in IOP at two years between the groups, although the trabeculectomy group required less IOP-lowering medication (25). Complications in the latter group were significantly higher. Comparing phacoemulsification to combined phacotrabeculectomy in medically uncontrolled PACG subjects, they found although the combined surgical group had lower IOP at 18 months, the phacoemulsification group still had a significant reduction in IOP (mean IOP at 18 months was 15.9mmHg) (26). Again, complications were higher in the combined surgical group. A similar study comparing phacoemulsification to phacotrabeculectomy in medically controlled PACG subjects found no difference in IOP between groups at 2 years (27).

 If significant PAS is present, combining phacoemulsification with goniosynechialysis (phaco-GSL) has yielded promising results, although all studies published to date have been case-series. In the first prospective series on this procedure, Teekhasaenee *et al*. found in a series of 52 eyes, 90.4% had IOP less than 20 mmHg without medications (28). In a more recent publication, Zhang *et al*. found that performing cataract extraction with visco-goniosynechialysis (phaco-VGSL) in subjects with medically uncontrolled IOP, mean IOP decreased from 45mmHg to 15 mmHg at 6 months in a series of 17 patients, without serious complication (29). These reductions are impressive for surgery which is relatively straightforward and which does not result in the creation of a filtration bleb. Indeed, Tang *et al*. compared phaco-VGSL to trabeculectomy in a retrospective study of 39 eyes and found that, at 10 months, IOP was lower in the phaco-VGSL group compared to the trabeculectomy group (14.1 vs 16.5 mmHg, p=0.025) (30). The concern with phaco-GSL has always been that the IOP lowering effects may be temporary. A publication from March this year from Japan however, found IOP less than 21mmHg in 85.9% of subjects undergoing phaco-GSL at 3 years, suggesting that the procedure may have long term success (31). In fact, it seems credible that long term success should be achievable, since, once the cataract is removed, it is unlikely that the drainage angle should narrow again post-procedure, since the peripheral anterior chamber deepens after cataract extraction (32). The group from Japan supplemented phaco-GSL with post-operative laser iridoplasty, and found that this led to significantly better results. This is an interesting adjunct and hopefully further publications will establish the safety and efficacy of this.

 These results suggest that in subjects with XFG or PACG, cataract surgery alone (especially if combined with GSL if indicated) can reduce IOP significantly (or adequately) in many cases. What about the largest group of patients seen in most glaucoma clinics, patients with POAG? The evidence that cataract surgery alone will reduce IOP significantly in these patients is weak or equivocal. Reviews from 2002 and 2010 reported that IOP decreasesaround 1.5 to 4.5mmHg after phacoemulsification in POAG subjects (1,33). The authors highlighted the importance of the statistical quirk known as ‘regression to the mean’ which may account for the supposed IOP reduction in many of the papers that were reviewed. Some clinicians have tried combining phacoemulsification surgery with endoscopic-cyclophotocoagulation (phaco-ECP). There have been a few studies in the last few years showing reasonable results with this procedure. In a retrospective analysis of 58 eyes from UK, IOP was reduced from 21.4mmHg to 14.4mmHg, which was significant (34). In another series from the UK of 63 eyes a similar reduction was found: 21.1mmHg to 15.1mmHg at 1 year and a significant reduction in the number of IOP-lowering medications (35). The results are moderately impressive although it is surprising that the procedure, with the technology having been around for so long, has not produced more data to support its effectiveness.

 If IOP is not lowered enough after phacoemulsification alone, selective laser trabeculoplasty (SLT) can be performed. This can sometimes result in significant lowering of IOP but the concern with SLT has always been that in some subjects there is minimal reduction and the effect of IOP lowering tends to decrease with time; although the laser can be repeated. SLT reduces IOP by approximately 18-40% (36), which may be sufficient in some patients. Several publications have examined the effectiveness of SLT in pseudophakic subjects with reduction of IOP of around 4mmHg or 25% depending on the study (37,38).

 The conclusion of this section is that cataract surgery alone (with GSL if need be) can certainly reduce IOP, sometimes to the low teens, particularly in subjects with XFG or PACG. Even if low teens IOPs are not achieved, it must be recognised that not all patients with glaucoma will require this level of IOP reduction. If cataract surgery alone (or combined with ECP) can reduce IOP to a degree, and even if IOP-lowering drops (or SLT) are necessary to lower IOP further, this still may be preferable to the patient having a trabeculectomy bleb. For those patients with more advanced glaucoma, or who need lower IOP, they may indeed require a subsequent trabeculectomy. If this is the case, at least a proportion of patients will be able to avoid having a trabeculectomy bleb. In addition, performing trabeculectomy in an eye which has already had cataract surgery may be preferable to performing trabeculectomy on a phakic eye, or performing combined phacotrabeculectomy. This will be discussed in the next section.

## If trabeculectomy is necessary, it is important to plan the surgery to give the best chance of success.

In many cases, of course, trabeculectomy surgery is necessary in order to achieve the low IOP levels that are needed to reduce the risk of further glaucomatous loss. It is important to operate under the conditions which will give the filtering bleb the best chance of maintaining a low IOP in the long term. As explained in the introduction, trabeculectomy is a half hour procedure and yet we expect its effectiveness to last for several years. The operation must therefore be performed under the most optimal conditions possible in order to ensure long-term success. In patients with cataract and medically uncontrolled glaucoma, the options are to perform trabeculectomy first then cataract surgery, cataract surgery first and then trabeculectomy, or to combine the surgery (phacotrabeculectomy). 

 In cases of XFG or PACG, the evidence suggests that it is preferable in most cases to perform cataract surgery first as, as has been shown in the section above. In many cases this will reduce IOP sufficiently so that glaucoma surgery is not necessary. In cases of PACG, the main cause for the high IOP is postulated to be obstruction of aqueous outflow due to a crowded anterior chamber. Taking out the cataract (or even the clear lens) will reduce this crowding and hence addresses the (probable) cause for the high IOP (32). When cataract surgery is performed, the surgeon should avoid making conjunctival incisions (e.g. sub-tenon’s anaesthesia) as this has been shown to increase subsequent trabeculectomy failure due to ‘priming’ of fibroblasts (39). This priming is thought to occur even if the conjunctival incisions are made away from the site of the subsequent trabeculectomy surgery. There are good clinical data also to support this idea. In a study from 1991, 66 subjects that had trabeculectomy were examined and risk factors for failure were entered into a multiple regression model. Formerconjunctival incisional surgery was associated with failure at 3 years (40). A later study by Broadway and Hitchings found success at 6 years after trabeculectomy was 93% in a control group versus 38% in a group that had had previous conjunctival incisional surgery (p<0.001) (41). Histological examination of the conjunctiva from the latter group found a significantly higher proportion of fibroblasts. Fontana *et al*. published two separate papers in 2006, which, if compared, have interesting findings. The first paper was a retrospective analysis of 89 eyes with open angle glaucoma which had had previous cataract surgery and then had trabeculectomy with MMC (42). The second paper was similar in design, but these subjects (292 eyes) had not had previous cataract surgery (43). Success based on IOP at 3 years was higher in the group that had trabeculectomy in phakic eyes. It should be noted that the group that had had previous cataract surgery, some of the surgery was performed by scleral tunnel (as opposed to clear corneal surgery) and this may be why the results were worse in the pseudophakic group. These studies imply that previous conjunctival incisional surgery does indeed have a negative impact on subsequent trabeculectomy. A more recent study found that, if trabeculectomy following clear corneal phacoemulsification surgery is compared to trabeculectomy in phakic eyes, success rates are similar (44).

 There is some evidence that prescribing a course of topical steroid medication prior to commencing trabeculectomy can reduce this priming (45). This has to be tempered with the fact that glaucoma patients are more prone to the steroid response and hence will need to be monitored for this. Furthermore, consideration should be given to prescribing NSAIDS or steroid medication less associated with the steroid response (e.g. g.fluorometholone or g.loteprednoletabonate 0.5%).

 However, it must be recognised that following cataract surgery, not all cases will achieve adequate IOP lowering and some may need glaucoma surgery subsequently. If this is necessary, it may be preferable to wait until the eye is stable before performing trabeculectomy. Cataract surgery causes significant amounts of inflammation in the anterior chamber, which is why steroid drops are prescribed routinely postoperatively. This inflammation, (presumably consisting of lens protein products, blood, breakdown of the blood aqueous barrier etc.,) could conceivably elicit more scarring if trabeculectomy is carried out soon after cataract surgery. Siriwardena *et al*. compared flare (using a flare meter) in the anterior chamber after phacoemulsification/IOL to flare after trabeculectomy (46). They found flare can last up to 6 months after routine phacoemulsification as opposed to one month after trabeculectomy. This implies that if trabeculectomy needs to be performed after cataract surgery, it may be preferable to wait 6 months, or maybe prescribe more intensive post-operative steroid drops. 

 In cases where there is minimal or no cataract, trabeculectomy surgery can be performed if the IOP is uncontrolled. The surgery will be on an eye which has had no previous surgical trauma and therefore should result in less fibrosis at the surgical site (compared to pseudophakic eyes for example). However, there is good evidence that trabeculectomy causes cataracts. A study from Singapore found 66% of subjects had a worsening of their lens opacities (by LOCS III units) at 3 years, and the majority of these occurred during the first year (47). Many other studies have found a similar association (48). The cause for increased cataracts after trabeculectomy remains unclearalthough a review by Mathew *et al*., postulated on some possible reasons for this association (49). As the evidence suggests that cataracts can develop or worsen soon after trabeculectomy, subsequent cataract surgery is a common occurrence. However, performing cataract surgery after trabeculectomy can negatively impact bleb function and this effect is increased the sooner cataract surgery is performed after trabeculectomy. The Singapore group, in a later publication, found approximate relative risk (RR) of bleb failure (defined as increased IOP) was 3.0 at 6 months, 1.7 at 1 year and 1.3 at 2 years (50). The implication of these findings is that if cataract surgery is performed in an eye that’s had a trabeculectomy, it may be preferable to wait at least a year, maybe longer, in order to reduce the risk of earlier bleb failure. There is good evidence that injecting anti-fibrotic agents (such as mitomycin-C (MMC), 5-fluorouracil (5-FU) or even bevacizumab )intraoperatively during cataract surgery may dampen the scarring process (51,52). Another reason to delay performing cataract surgery after trabeculectomy is that in terms of bleb features such as bleb height, vascularity and presence of microcysts, there is evidence that these features vary considerable over the first 6 months post-trabeculectomy, after which a greater degree of stability occurs (53). It is tempting to attribute this to the bleb remodelling during the first 6 months and therefore retains some degree of plasticity. Any trauma (iatrogenic otherwise) during this period of plasticity is likely to be detrimental to the bleb remodelling process. This correlates well with clinical experience in which the first 3 to 6 months, patients are monitored frequently and bleb manipulations are frequently carried out. After 6 months, this occurs much less frequently.

 The convenience of performing cataract and trabeculectomy surgery together has made phacotrabeculectomyvery popular with many surgeons. But one has to ask is the convenience (for both patient and surgeon) worth it if it has a negative impact in the long term lowering effectiveness of the trabeculectomy part of the procedure? To see if there is indeed a negative impact with phacotrabeculectomy, one needs to examine the published data and, if this is lacking, examine the procedure from the principle of biological plausibility. 

 Looking at the data first, there have been few large; long-term prospective studies comparing trabeculectomy alone (either in phakic or pseudophakic eyes) to combined phacotrabeculectomy, although more are needed, given the popularity of these procedures worldwide. In a prospective study a group of 44 subjects of elderly white patients with POAG had phacotrabeculectomy and were matched to a group that had trabeculectomy. The trabeculectomy group were significantly more successful and effectivethan the phacotrabeculectomy group (54). 

 All the surgeries were unaugmented. In a more recent prospective study from Japan comparing the 2 groups who all had surgery augmented with MMC, the authors also found the trabeculectomy group had significantly better success, although the study was only for 1 year (55). Kleinmann *et al*. found similar results in a retrospective comparative study of 102 MMC-augmented eyes, with significantly greater reduction in IOP in the trabeculectomy group (56). There have been smaller, retrospective studies. Singh *et al*. published a retrospective study of 102 eyes (51 in each group) comparing trabeculectomy and phacotrabeculectomy (both with post-operative 5-FU injections) and found success (defined as IOP more than 16 mmHg at 2 years) to be 71% on the trabeculectomy group compared to 55% in the phacotrabeculectomy group (p<0.01) (57). Chang *et al*. performed a similar study comparing the 2 groups, although this time intraoperative 5-FU was used in both groups (58). Although success at 6 years was similar between the groups, there were differences between the groups. The trabeculectomy group had significantly higher per-operative IOP, was significantly younger and had significantly less postoperative 5-FU injections compared to the phacotrabeculectomy group. In terms of the percentage drop in IOP, it was significantly higher in the trabeculectomy alone group compared to the combined surgery group (44.6% vs. 31.2%, p=0.02). There is some evidence to the contrary. Vernon’s group published a retrospective study of 37 eyes and compared those that had had microtrabeculectomy to those that had phaco-microtrabeculectomy and found no difference at 3 years in IOP (59). Murthy *et al*. also retrospectively compared trabeculectomy to phacotrabeculectomy and found that, at 2 years, IOP was not significantly different, although the trabeculectomy group had a greater reduction in IOP compared to the combined surgery group (60), similar to the results of the study by Derick *et al*. in 1998 (61).

 As can be seen, the data for superiority of trabeculectomy over phacotrabeculectomy (or vice versa) in terms of IOP control is weak. In these cases, it is useful to deconstruct the procedures to determine if anything about them could give us an idea as to which one is more likely to be successful from a biological plausibility perspective. The main difference is that phacotrabeculectomy involves removal of the crystalline lens at the same time as the trabeculectomy. We know that cataract removal results in anterior chamber inflammation, and that this can last up to 6 months (46). We also know that the earlier one performs cataract surgery after trabeculectomy, the earlier trabeculectomy failure occurs (RR of failure if phacoemulsification is performed 6 months after trabeculectomy is 3.0, in contrast to a RR of 1.3 if the time between surgeries is 2 years) (50). It could therefore be argued that if the time between trabeculectomy and cataract surgery is zero (as in combined phacotrabeculectomy), this ought to lead to earlier failure. This would presumably be because the inflammatory material as a result of the phacoemulsification (lens proteins etc.) would immediately be passing through the sclerostomy and into the sub-tenons space during the early post-operative period–precisely the time when the most fibrosis at the trabeculectomy site occurs. It has to be emphasised again that high quality clinical data for earlier failure in phacotrabeculectomy are lacking, but from a biological plausibility point of view, this hypothesis does have some credibility.

## Practical guide on how to manage patients with cataract and medically uncontrolled glaucoma

Based on the 3 principles described in the introduction, for which some justification has been provided in the sections above, a management strategy for patients with cataract and medically uncontrolled glaucoma can be extrapolated.

 In almost all cases (see below for exceptions), if there is significant cataract (defined qualitatively), it would be preferable to perform cataract surgery first, using a clear corneal tunnel and avoiding making conjunctival incisions. The advantage of this approach is clear for subjects with XFG or PACG, in that significant IOP lowering can sometimes be achieved. In cases with PACG, if there are more than 90 degrees of PAS, goniosynechialysis can be performed at the same time as the cataract surgery. Even in cases of POAG, it is still worth removing the cataract first as this may result in IOP lowering in a proportion of cases. Those cases in which the IOP is not adequately lowered may need subsequent trabeculectomy, although it is advisable to wait 6 months before this is performed, if possible. Oral acetazolamide may be considered as a temporizing measure. It is good practice to inform the patient that further glaucoma surgery may be necessary if indicated, during the consent process for the cataract surgery. Another advantage of performing cataract surgery first (as opposed to phacotrabeculectomy) is that, especially for subjects with PXF or PACG, the cataract surgery itself can be difficult, involving iris hooks for example, or increasing complication rates. It is far better to manage these cataracts separately rather than in conjunction with a trabeculectomy. 

 If there is minimal or no cataract, for patients with POAG, trabeculectomy can be performed. Again, it is advisable to inform the patient that subsequent cataract surgery may be necessary, and if so, it may be preferable to delay such surgery for at least a year, to allow the bleb morphology to stabilise and to reduce the risk of earlier bleb failure. Any cataract surgery, if it is performed, ought to be augmented with MMC/5-FU or bevacizumab. 

For cases of PXF or PACG and no lens opacity, it is debatable if cataract surgery should be performed before the trabeculectomy. The EAGLE study, the results of which will be available soon, will help answer this question for cases of PACG (62). In most cases, however, it would still be advisable to perform cataract surgery first, even if there is no cataract. For XFG, the longer one waits before performing cataract surgery, the more likely it is to encounter problems such as poor dilation and zonular instability. In addition, the data suggests that removing the lens can reduce the risk of further IOP rises, as discussed previously. For subjects with PACG and no cataract, the problem with performing trabeculectomy first is that trabeculectomy results in a shallowing of the anterior chamber and this shallowing remains even at 5 years post-procedure (9). If one accepts that at least part of the mechanism for IOP rise in PACG is a crowded anterior chamber leading to irido-trabecular contact, then trabeculectomy alone does not address this problem, indeed, it will exacerbate it. Taking out the cataract addresses the problem of a crowded anterior chamber and may lower IOP in many cases. It is safer to perform a trabeculectomy in a pseudophakic eye than in a phakic eye with a shallow anterior chamber. Complications such as aqueous misdirection would be more likely in the latter scenario.

 It seems there is little indication or benefit in performing combined phacotrabeculectomy. However, this view probably over-emphasises the theoretical disadvantages of this procedure. One scenario where phacotrabeculectomy might be the procedure of choice is in patients with high IOP, cataract and advanced glaucoma (particularly open angle). In these patients, performing cataract surgery first may result in a pressure spike which could result in disc ‘wipe-out’ (63). Low IOP at all points in the post-operative period is imperative and likely to be achieved only with combined surgery. Also, in such a patient it is very likely that trabeculectomy will be needed to achieve the low target pressure and the luxury of waiting 6 months after cataract surgery before performing trabeculectomy is not likely to be available. Another scenario is if the patient is unable to have 2 separate surgeries, due to financial or other constraints. Elderly patients with short life-expectancy may also fall in to this group although it is dangerous to predict life-expectancy based solely on chronological age.

## Summary

Before trabeculectomy surgery is performed, it is important that the implications of the surgery are thoroughly understood particularly in the long term. Other, more straightforward procedures should be considered (such as cataract surgery) and a decision based on that individual patient, based on age, severity of visual field loss, intraocular pressure, rate of glaucomatous progression, type of glaucoma and amount of cataract needs to be made. In most cases, cataract surgery with or without additional procedures such as GSL or ECP should be carried out and such surgery should avoid conjunctival incisions. For those subjects that go on to require trabeculectomy, it’s best to wait at least 6 months. For patients with POAG and no cataract, trabeculectomy can be performed fist, and if subsequent cataract surgery is needed, best to wait for at least a year and then augment with surgery with an anti-fibrotic agent. Combined phacotrabeculectomy should be reserved for subjects with high IOP, advance glaucoma and cataract or how are unable or unwilling to undergo separate procedures. Other techniques to reduce scarring during and after trabeculectomy (e.g. antimetabolite use, use of releasable sutures, etc.) need due consideration also of course to ensure long term IOP control, but these are beyond the scope of this review. At all times patients need to kept informed of the management decisions with appropriate informed consent taken. It is also advisable that they are ‘warned’ of future procedures that may be necessary in order to prepare them mentally. It is hoped that by following this management strategy long term IOP control will become more likely and the side effects or complications of treatment will be minimised.

## References

[1] Friedman DS, Jampel HD, Lubomski LH, Kempen JH, Quigley H, Congdon N, Levkovitch-Verbin H, Robinson KA, Bass EB (2002). Surgical strategies for coexisting glaucoma and cataract: an evidence-based update. Ophthalmology.

[2] Verges C, Cazal J, Lavin C (2005). Surgical strategies in patients with cataract and glaucoma. CurrOpinOphthalmol.

[3] Vass C, Menapace R (2004). Surgical strategies in patients with combined cataract and glaucoma. CurrOpinOphthalmol.

[4] Vizzeri G, Weinreb RN (2010). Cataract surgery and glaucoma. CurrOpinOphthalmol.

[5] Husain R, Clarke JC, Seah SK, Khaw PT (2005). A review of trabeculectomy in East Asian people--the influence of race. Eye (Lond).

[6] Nakano Y, Araie M, Shirato S (1989). Effect of postoperative subconjunctival 5-fluorouracil injections on the surgical outcome of trabeculectomy in the Japanese. Graefes Arch ClinExpOphthalmol.

[7] Johnson DH, Matsumoto Y (2000). Schlemm's canal becomes smaller after successful filtration surgery. Arch Ophthalmol.

[8] Dietlein TS, Lüke C, Jacobi PC, Krieglstein GK (2002). Individual factors influencing trabecular morphology in glaucoma patients undergoing filtration surgery. J Glaucoma.

[9] Husain R, Li W, Gazzard G, Foster PJ, Chew PT, Oen FT, Phillips R, Khaw PT, Seah SK, Aung T (2013). Longitudinal changes in anterior chamber depth and axial length in Asian subjects after trabeculectomy surgery. Br J Ophthalmol.

[10] Zahid S, Musch DC, Niziol LM, Lichter PR (2013). Collaborative Initial Glaucoma Treatment Study Group Risk of Endophthalmitis and Other Long-Term Complications of Trabeculectomy in the Collaborative Initial Glaucoma Treatment Study (CIGTS). Am J Ophthalmol.

[11] Rai P, Kotecha A, Kaltsos K, Ruddle JB, Murdoch IE, Bunce C, Barton K (2012). Changing trends in the incidence of bleb-related infection in trabeculectomy. Br J Ophthalmol.

[12] Mac I, Soltau JB (2003). Glaucoma-filtering bleb infections. CurrOpinOphthalmol.

[13] Bindlish R, Condon GP, Schlosser JD, D'Antonio J, Lauer KB, Lehrer R (2002). Efficacy and safety of mitomycin-C in primary trabeculectomy: five-year follow-up. Ophthalmology.

[14] Budenz DL, Hoffman K, Zacchei A (2001). Glaucoma filtering bleb dysesthesia. Am J Ophthalmol.

[15] Janz NK, Wren PA, Lichter PR, Musch DC, Gillespie BW, Guire KE (2001). Quality of life in newly diagnosed glaucoma patients : The Collaborative Initial Glaucoma Treatment Study. Ophthalmology.

[16] Jampel HD, Musch DC, Gillespie BW, Lichter PR, Wright MM, Guire KE (2005). Collaborative Initial Glaucoma Treatment Study Group Perioperative complications of trabeculectomy in the collaborative initial glaucoma treatment study (CIGTS). Am J Ophthalmol.

[17] Shue A, Joseph JM, Tao JP (2013). Repair of Eyelid Retraction due to a Trabeculectomy Bleb: Case Series and Review of the Literature. OphthalPlastReconstr Surg.

[18] Vesti E, Kivelä T (2000). Exfoliation syndrome and exfoliation glaucoma. ProgRetin Eye Res.

[19] Ritch R, Schlötzer-Schrehardt U (2001). Exfoliation syndrome. SurvOphthalmol.

[20] Damji KF, Konstas AG, Liebmann JM, Hodge WG, Ziakas NG, Giannikakis S, Mintsioulis G, Merkur A, Pan Y, Ritch R (2006). Intraocular pressure following phacoemulsification in patients with and without exfoliation syndrome: a 2 year prospective study. Br J Ophthalmol.

[21] Dinslage S, Rosentreter A, Schild AM, Jordan JF, Widder R, Dietlein TS (2012). Combined phacoemulsification with trabecular aspiration with differing outcomes in pseudoexfoliative glaucoma - a retrospective study. Klin Monbl Augenheilkd.

[22] Georgopoulos GT, Chalkiadakis J, Livir-Rallatos G, Theodossiadis PG, Theodossiadis GP (2000). Combined clear cornea phacoemulsification and trabecular aspiration in the treatment of pseudoexfoliative glaucoma associated with cataract. Graefes Arch Clin Exp Ophthalmol.

[23] Shingleton BJ, Laul A, Nagao K, Wolff B, O'Donoghue M, Eagan E, Flattem N, Desai-Bartoli S (2008). Effect of phacoemulsification on intraocular pressure in eyes with pseudoexfoliation: single-surgeon series. J Cataract Refract Surg.

[24] Shingleton BJ, Wooler KB, Bourne CI, O'Donoghue MW (2011). Combined cataract and trabeculectomy surgery in eyes with pseudoexfoliation glaucoma. J Cataract Refract Surg.

[25] Tham CC, Kwong YY, Baig N, Leung DY, Li FC, Lam DS (2013). Phacoemulsification versus Trabeculectomy in Medically Uncontrolled Chronic Angle-Closure Glaucoma without Cataract. Ophthalmology.

[26] Tham CC, Kwong YY, Leung DY, Lam SW, Li FC, Chiu TY, Chan JC, Lam DS, Lai JS (2009). Phacoemulsification versus combined phacotrabeculectomy in medically uncontrolled chronic angle closure glaucoma with cataracts. Ophthalmology.

[27] Tham CC, Kwong YY, Leung DY, Lam SW, Li FC, Chiu TY, Chan JC, Chan CH, Poon AS, Yick DW, Chi CC, Lam DS, Lai JS (2008). Phacoemulsification versus combined phacotrabeculectomy in medically controlled chronic angle closure glaucoma with cataract. Ophthalmology.

[28] Teekhasaenee C, Ritch R (1999). Combined phacoemulsification and goniosynechialysis for uncontrolled chronic angle-closure glaucoma after acute angle-closure glaucoma. Ophthalmology.

[29] Zhang Z, Chen M, Yin J, Yao K (2012). Microcoaxial Phacoemulsification Combined With Viscogoniosynechialysis for Patients With Refractory Acute Angle-closure Glaucoma. J Glaucoma.

[30] Tang Y, Qian S, Wang J, Yao J, Xu J, Cheng L, Zhou C (2012). Effects of combined phacoemulsification and viscogoniosynechialysis versus trabeculectomy in patients with primary angle-closure glaucoma and coexisting cataract. Ophthalmologica.

[31] Kameda T, Inoue T, Inatani M, Tanihara H (2013). Japanese Phaco-Goniosynechialysis Multicenter Study Group Long-term efficacy of goniosynechialysis combined with phacoemulsification for primary angle closure. Graefes Arch ClinExpOphthalmol.

[32] Latifi G, Moghimi S, Eslami Y, Fakhraie G, Zarei R, Lin S (2012). Effect of phacoemulsification on drainage angle status in angle closure eyes with or without extensive peripheral anterior synechiae. Eur J Ophthalmol.

[33] Shrivastava A, Singh K (2010). The effect of cataract extraction on intraocular pressure. CurrOpinOphthalmol.

[34] Lindfield D, Ritchie RW, Griffiths MF (2012). 'Phaco-ECP': combined endoscopic cyclophotocoagulation and cataract surgery to augment medical control of glaucoma. BMJ Open.

[35] Clement CI, Kampougeris G, Ahmed F, Cordeiro MF, Bloom PA (2013). Combining phacoemulsification with endoscopic cyclophotocoagulation to manage cataract and glaucoma. Clin Experiment Ophthalmol.

[36] Barkana Y, Belkin M (2007). Selective laser trabeculoplasty. Surv Ophthalmol.

[37] Rosenfeld E, Shemesh G, Kurtz S (2012). The efficacy of selective laser trabeculoplasty versus argon laser trabeculoplasty in pseudophakic glaucoma patients. Clin Ophthalmol.

[38] Shazly TA, Latina MA, Dagianis JJ, Chitturi S (2011). Effect of prior cataract surgery on the long-term outcome of selective laser trabeculoplasty. Clin Ophthalmol.

[39] Broadway DC, Chang LP (2001). Trabeculectomy, risk factors for failure and the preoperative state of the conjunctiva. J Glaucoma.

[40] Miller MH, Rice NS (1991). Trabeculectomy combined with beta irradiation for congenital glaucoma. Br J Ophthalmol.

[41] Broadway DC, Grierson I, Hitchings RA (1998). Local effects of previous conjunctival incisional surgery and the subsequent outcome of filtration surgery. Am J Ophthalmol.

[42] Fontana H, Nouri-Mahdavi K, Caprioli J (2006). Trabeculectomy with mitomycin C in pseudophakic patients with open-angle glaucoma: outcomes and risk factors for failure. Am J Ophthalmol.

[43] Fontana H, Nouri-Mahdavi K, Lumba J, Ralli M, Caprioli J (2006). Trabeculectomy with mitomycin C: outcomes and risk factors for failure in phakic open-angle glaucoma. Ophthalmology.

[44] Supawavej C, Nouri-Mahdavi K, Law SK, Caprioli J (2013). Comparison of results of initial trabeculectomy with mitomycin c after prior clear-corneal phacoemulsification to outcomes in phakic eyes. J Glaucoma.

[45] Broadway DC, Grierson I, Stürmer J, Hitchings RA (1996). Reversal of topical antiglaucoma medication effects on the conjunctiva. Arch Ophthalmol.

[46] Siriwardena D, Kotecha A, Minassian D, Dart JK, Khaw PT (2000). Anterior chamber flare after trabeculectomy and after phacoemulsification. Br J Ophthalmol.

[47] Husain R, Aung T, Gazzard G, Foster PJ, Devereux JG, Chew PT, Oen FT, Khaw PT, Seah SK (2006). Effect of trabeculectomy on lens opacities in an East Asian population. Arch Ophthalmol.

[48] Hylton C, Congdon N, Friedman D, Kempen J, Quigley H, Bass E, Jampel H (2003). Cataract after glaucoma filtration surgery. Am J Ophthalmol.

[49] Mathew RG, Murdoch IE (2011). The silent enemy: a review of cataract in relation to glaucoma and trabeculectomy surgery. Br J Ophthalmol.

[50] Husain R, Liang S, Foster PJ, Gazzard G, Bunce C, Chew PT, Oen FT, Khaw PT, Seah SK, Aung T (2012). Cataract surgery after trabeculectomy: the effect on trabeculectomy function. Arch Ophthalmol.

[51] Sharma TK, Arora S, Corridan PG (2007). Phacoemulsification in patients with previous trabeculectomy: role of 5-fluorouracil. Eye (Lond).

[52] Coote MA, Ruddle JB, Qin Q, Crowston JG (2008). Vascular changes after intra-bleb injection of bevacizumab. J Glaucoma.

[53] Husain R (2010). A comparative study of the natural history of unaugmented and 5-fluorouracil augmented glaucoma filtration surgery in an East Asian population. Thesis.

[54] Lochhead J, Casson RJ, Salmon JF (2003). Long term effect on intraocular pressure of phacotrabeculectomy compared to trabeculectomy. Br J Ophthalmol.

[55] Ogata-Iwao M, Inatani M, Takihara Y, Inoue T, Iwao K, Tanihara H (2013). A prospective comparison between trabeculectomy with mitomycin C and phacotrabeculectomy with mitomycin C. ActaOphthalmol.

[56] Kleinmann G, Katz H, Pollack A, Schechtman E, Rachmiel R, Zalish M (2002). Comparison of trabeculectomy with mitomycin C with or without phacoemulsification and lens implantation. Ophthalmic Surg Lasers.

[57] Singh RP, Goldberg I, Mohsin M (2001). The efficacy and safety of intraoperative and/or postoperative 5-fluorouracil in trabeculectomy and phacotrabeculectomy. Clin Experiment Ophthalmol.

[58] Chang L, Thiagarajan M, Moseley M, Woodruff S, Bentley C, Khaw PT, Bloom P (2006). Intraocular pressure outcome in primary 5FU phacotrabeculectomies compared with 5FU trabeculectomies. J Glaucoma.

[59] Rotchford AP, Vernon SA (2007). Phaco-microtrabeculectomy: technique and intraocular pressure control in comparison with microtrabeculectomy. Clin Experiment Ophthalmol.

[60] Murthy SK, Damji KF, Pan Y, Hodge WG (2006). Trabeculectomy and phacotrabeculectomy, with mitomycin-C, show similar two-year target IOP outcomes. Can J Ophthalmol.

[61] Derick RJ, Evans J, Baker ND (1998). Combined phacoemulsification and trabeculectomy versus trabeculectomy alone: a comparison study using mitomycin-C. Ophthalmic Surg Lasers.

[62] Azuara-Blanco A, Burr JM, Cochran C, Ramsay C, Vale L, Foster P, Friedman D, Quayyum Z, Lai J, Nolan W, Aung T, Chew P, McPherson G, McDonald A, Norrie J (2011). Effectiveness in Angle-closure Glaucoma of Lens Extraction (EAGLE) Study Group The effectiveness of early lens extraction with intraocular lens implantation for the treatment of primary angle-closure glaucoma (EAGLE): study protocol for a randomized controlled trial. Trials.

[63] Aggarwal SP, Hendeles S (1986). Risk of sudden visual loss following trabeculectomy in advanced primary open-angle glaucoma. Br J Ophthalmol.

